# IDP-Head: An Interactive Dual-Perception Architecture for Organoid Detection in Mouse Microscopic Images

**DOI:** 10.3390/biomimetics10090614

**Published:** 2025-09-11

**Authors:** Yuhang Yang, Changyuan Fan, Xi Zhou, Peiyang Wei

**Affiliations:** 1 School of Software, Xinjiang University, Urumqi 830091, China; 107552305010@stu.xju.edu.cn; 2 College of Electronic Engineering, Chengdu University of Information Technology, Chengdu 610225, China; 3 The Xinjiang Technical Institute of Physics and Chemistry, Chinese Academy of Sciences, Urumqi 830011, China; zhouxi@ms.xjb.ac.cn; 4 School of Software Engineering, Chengdu University of Information Technology, Chengdu 610225, China; weipy@cuit.edu.cn; 5 Key Laboratory of Remote Sensing Application and Innovation, Chongqing 401147, China; 6 Dazhou Key Laboratory of Government Data Security, Sichuan University of Arts and Science, Dazhou 635000, China

**Keywords:** organoids, biological structures, quantitative analysis, IDP-Head

## Abstract

The widespread application of organoids in disease modeling and drug development is significantly constrained by challenges in automated quantitative analysis. In bright-field microscopy images, organoids exhibit complex characteristics, including irregular morphology, blurred boundaries, and substantial scale variations, largely stemming from their dynamic self-organization that mimics in vivo tissue development. Existing convolutional neural network-based methods are limited by fixed receptive fields and insufficient modeling of inter-channel relationships, making them inadequate for detecting such evolving biological structures. To address these challenges, we propose a novel detection head, termed Interactive Dual-Perception Head (IDP-Head), inspired by hierarchical perception mechanisms in the biological visual cortex. Integrated into the RTMDet framework, IDP-Head comprises two bio-inspired components: a Large-Kernel Global Perception Module (LGPM) to capture global morphological dependencies, analogous to the wide receptive fields of cortical neurons, and a Progressive Channel Synergy Module (PCSM) that models inter-channel semantic collaboration, echoing the integrative processing of multi-channel stimuli in neural systems. Additionally, we construct a new organoid detection dataset to mitigate the scarcity of annotated data. Extensive experiments on both our dataset and public benchmarks demonstrate that IDP-Head achieves a 5-percentage-point improvement in mean Average Precision (mAP) over the baseline model, offering a biologically inspired and effective solution for high-fidelity organoid detection.

## 1. Introduction

The advent of three-dimensional (3D) organoid technology marks a paradigm shift in life sciences and regenerative medicine [1]. These stem cell-derived cultures, which faithfully recapitulate key structures and functions of their corresponding organs [2,3], offer unprecedented opportunities for research in areas such as disease modeling and high-throughput drug screening [4,5]. However, translating the immense potential of this technology into reliable experimental conclusions and novel biological findings is critically limited by the need for precise and efficient automated phenotypic quantification of vast amounts of microscopy images. This task is particularly challenging due to the inherent complexities of organoid images [6]: during dynamic cultivation, organoids exhibit high morphological heterogeneity [7], indistinct tissue boundaries, and substantial scale variations. Among these, scale variation stands out as one of the most prominent and driving challenges. Such proportional differences are not only reflected in the size disparities across different samples, but also manifest throughout the continuous deformation and growth of the same organoid during its developmental and dynamic evolutionary processes. These complex variations significantly compromise the stability of object detection and feature extraction, posing severe difficulties for existing visual recognition methods. Therefore, effectively addressing scale variation should be regarded as a core design motivation in the development of automated analysis systems, as it plays a decisive role in enhancing model robustness, generalization ability, and practical applicability.

Although deep learning, particularly Convolutional Neural Networks (CNNs), has become a mainstream technology for biomedical image analysis [8], existing object detectors—whether two-stage frameworks like Faster R-CNN [9] or one-stage ones like the YOLO [10] series—face two fundamental constraints when directly applied to organoid detection. The first lies in the limitation of spatial representation: the local computational paradigm of standard convolutional kernels inherently restricts their effective receptive field, making it difficult to capture the long-range spatial dependencies necessary for large, irregular organoids. The second is the bottleneck in modeling channel-wise semantics: while methods like the seminal SENet introduce channel attention, their reliance on linear fully-connected layers is insufficient for capturing the inherent higher-order, non-linear inter-channel correlations present in complex biological images. Departing from conventional detectors based on Convolutional Neural Networks (CNNs), the field of visual recognition, particularly in biomedical imaging, has witnessed the rise of novel architectures such as pure Transformers and hybrid convolutional-attention models. The key strength of these architectures is their self-attention mechanism, which empowers them to model long-range spatial dependencies and intricate inter-channel relationships, thereby overcoming the inherent locality bias of traditional convolutions. Pioneering technologies like Vision Transformer (ViT) and its hierarchical variant, Swin Transformer, exemplify the significant potential of this approach [11].

It is noteworthy that most current improvements in object detection focus on the network’s backbone or feature pyramid layers [12]. However, the internal feature enhancement design of the detection head—the pivotal component that translates high-level semantic features [13] into final predictions (classes and bounding boxes)—remains a largely overlooked research gap. To fill this critical gap, our work draws inspiration from the remarkable efficiency of the human visual system. We design a novel Interactive Dual-Perception Head (IDP-Head), founded on a biomimetic computational paradigm, and seamlessly integrate it into the RTMDet framework [14]. Its core idea is to reshape and enhance input features through two synergistic modules designed to emulate biological perception mechanisms before they are passed to the classification and regression branches [15]. We propose the Large-Kernel Global Perception Module (LGPM) and the Progressive Channel Synergy Module (PCSM) to enhance feature representation for organoid analysis. Inspired by the perceptual mechanism of the visual cortex for holistic scenes, the LGPM establishes long-range spatial dependencies to overcome the limited receptive fields of conventional convolutions, enabling the precise capture of macroscopic organoid structures. Concurrently, the PCSM emulates the brain’s approach to integrating multi-channel information. It models non-linear inter-channel relationships and adaptively enhances salient features via a learnable weighting parameter, thereby improving the model’s recognition accuracy for small-scale or low-contrast organoids [16].

The main contributions of this study are as follows:

We propose the LGPM module to address the challenge of complex organoid morphology. This module effectively captures long-range spatial dependencies, enabling the model to perceive the holistic structure of irregularly shaped organoids and significantly improving detection accuracy under high morphological variance.

We design the PCSM to achieve precise localization of organoids with indistinct boundaries. By adaptively modeling non-linear inter-channel relationships, the PCSM enhances crucial semantic features while suppressing background noise, thereby reducing ambiguity and improving detection accuracy in low-contrast bright-field images.

We construct a comprehensive, well-annotated organoid detection benchmark dataset. This provides reliable data support for quantitative studies of organoids and facilitates reproducible evaluations and method comparisons.

Experimental results on two datasets demonstrate that the proposed method performs competitively on key metrics such as mAP, underscoring its robustness and ability to generalize.

The remainder of this paper is organized as follows. Section 2 reviews the applications and developments of object detection techniques in the field of medical imaging. Section 3 elaborates on the experimental materials and our proposed methodology, with its core components being the IDP-Head and the Multi-branch Prediction module. Section 4 presents the overall experimental design, including the setup for comparative experiments and ablation studies, as well as the corresponding evaluation metrics and results. Section 5 provides an in-depth discussion and analysis of the experimental results, covering the interpretation of key metrics such as the confusion matrix, precision, and recall. Finally, Section 6 concludes this paper by summarizing our research work and provides an outlook on future research directions.

## 2. Related Work

This section reviews the relevant literature from two primary aspects. We first elucidate the performance bottlenecks of generic object detection methods when applied to the analysis of dense organoid images. Subsequently, by examining advanced visual perception techniques, we observe that the majority of prior research has focused on optimizing the network backbone, thereby highlighting a critical research gap in the design of the detection head.

### 2.1. Application of Object Detection in Medical Image Analysis

Early medical image analysis relied on conventional image processing techniques [17], such as thresholding and morphological operations [18]. However, these methods exhibit limited robustness and generalization capabilities when faced with the complexity and diversity of organoid images. Consequently, object detection [19] frameworks based on deep learning have become the mainstream paradigm in this field. Two-stage detectors, represented by Faster R-CNN, excel in tasks requiring high precision, like lesion detection, but their significant computational overhead limits their application in high-throughput screening scenarios. For this reason, one-stage detectors, such as the YOLO series and RTMDet, are favored for their exceptional efficiency. Nevertheless, when directly applied to dense organoid culture environments, these general-purpose frameworks often fail to achieve robust and accurate detection due to challenges like severe object overlap [20], dramatic scale variations, and blurred boundaries [21]. Fundamentally, their performance bottleneck lies in the fact that the design of their core feature extraction and perception modules is not fully adapted to the unique challenges of biological imagery.

### 2.2. Advanced Visual Perception Mechanisms and the Research Gap in Detection Head Design

To enhance feature representation, researchers have explored various advanced visual perception mechanisms from two primary dimensions: spatial and channel. In the spatial dimension, large-kernel convolution designs, represented by recent developments like ConvNeXt and RepLKNet [22], have demonstrated potential, surpassing traditional spatial attention. They can effectively expand the receptive field to capture long-range dependencies while maintaining computational efficiency. In the channel dimension, channel attention mechanisms [23], led by the seminal SENet [24], enhance key features by explicitly modeling inter-channel correlations. However, their simplified linear modeling approach struggles to capture complex non-linear semantics. Notably, a crucial point that is often overlooked is that most of these advanced perception mechanisms are applied to the network backbone [25]. Consequently, the detection head—the “last mile” responsible for translating high-level features into final predictions—remains deficient in designs for synergistic enhancement.

## 3. Materials and Methods

### 3.1. Datasets

**Figure 1:**
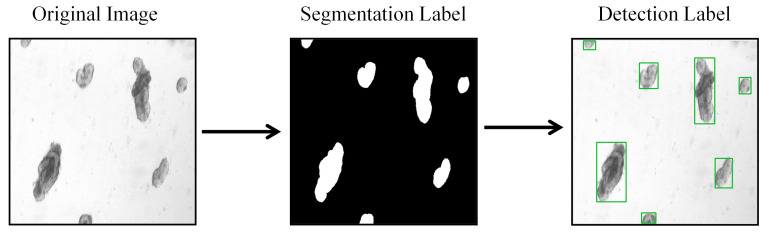
Data Transformation Process of MouseOrg.

**Figure 2:**
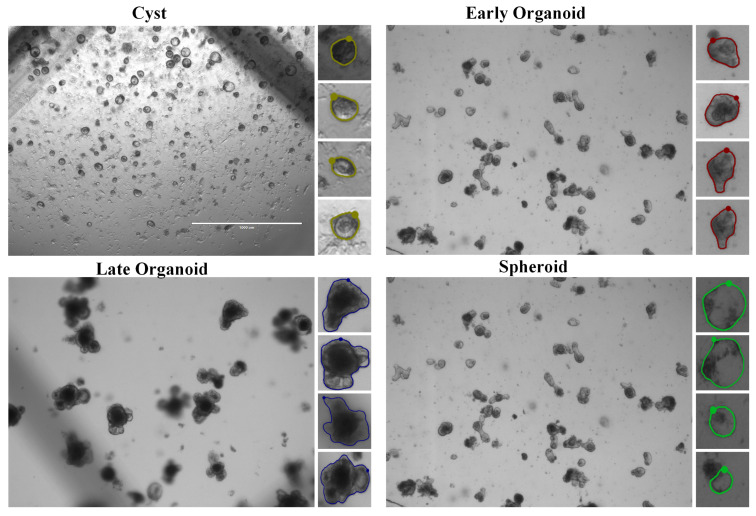
Categorical Overview of Tellorg Dataset.

To comprehensively evaluate our proposed method, we utilized two mouse organoid datasets with distinct characteristics and challenges: Mouse2Org and TelluOrg. The first, Mouse2Org, is a binary-class detection dataset containing 508 images [26], with its annotations converted from a semantic segmentation project; this conversion process is illustrated in Figure 1. In contrast, the TelluOrg dataset introduces a higher level of complexity, comprising 840 bright-field microscopy images that [27] encompass four distinct developmental stages of organoids, examples of which are shown in Figure 2. All the organoids in this dataset originated from crypts isolated from whole mouse small intestines. Based on clear morphological features, these organoids were manually classified into four categories: Cyst, defined as vesicular structures without any budding; Early Organoid, which has formed one to two crypts; Late Organoid, exhibiting three or more mature budding structures; and Spheroid, an independent spherical category with no apparent crypts. The TelluOrg dataset contains a total of 23,066 annotated instances. Together, these two datasets form a comprehensive evaluation benchmark, spanning from basic binary-class detection to complex, imbalanced, multi-class scenarios.

### 3.2. Description of Compared Models

To comprehensively validate the effectiveness and advancement of our proposed method, Org-IDPNet, we selected a diverse and representative range of mainstream object detectors for comparison, ensuring both breadth and depth in our evaluation. The compared methods span multiple paradigms: from the classic two-stage detector Faster R-CNN to balanced single-stage models such as TOOD and CenterNet; from Transformer-based architectures that are a current research hotspot, like Deformable-DETR and DINO, to the efficient lightweight designs represented by EfficientDet and YOLOv11; and even emerging detection paradigms, as exemplified by DiffusionDet. Furthermore, we conduct a direct comparison between our model and RTMDet-L. As our baseline model, this comparison most directly demonstrates the performance improvement brought by our proposed IDP-Head.

### 3.3. Evaluation Metrics

To provide a comprehensive and rigorous assessment of our model’s performance, we employed a suite of standard metrics widely recognized in the field of object detection. The evaluation hinges on the concepts of precision and recall, which are defined as follows:

(1) $$\mathrm{Precision}=\frac{\mathrm{TP}}{\mathrm{TP}+\mathrm{FP}}$$

(2) $$\mathrm{Recall}=\frac{\mathrm{TP}}{\mathrm{TP}+\mathrm{FN}}$$

where TP (True Positives), FP (False Positives), and FN (False Negatives) are determined based on the Intersection over Union (IoU) between the predicted bounding box ($B_{p}$) and the ground-truth box ($B_{gt}$). The IoU is calculated as follows:

(3) $$\mathrm{IoU}=\frac{\mathrm{area}(B_{p}\cap B_{gt})}{\mathrm{area}(B_{p}\cup B_{gt})}$$

The primary metric for accuracy is mean Average Precision (mAP). For a single object class, the Average Precision (AP) is computed by calculating the area under the precision–recall curve, which can be expressed as follows:

(4) $$\mathrm{AP}=\int_{0}^{1}p\left(r\right)\;dr$$

where p(r) is the precision as a function of the recall *r*. The mAP is then the mean of AP values over all *C* object classes:

(5) $$\mathrm{mAP}=\frac{1}{C}\sum_{i=1}^{C}{\mathrm{AP}}_{i}$$

In line with standard benchmarks, our principal reported metric is the mean AP calculated over a range of IoU thresholds from 0.5 to 0.95 with a step size of 0.05 (often denoted as mAP [0.5:0.95]). Additionally, to provide a more nuanced view, we report the AP at a lenient threshold, mAP_50_ (IoU = 0.5), and at a strict threshold, mAP_75_ (IoU = 0.75), which better reflects the model’s precise localization capability. To further evaluate the model’s adaptability to object size variations, we analyze its performance on small (mAP_S_, medium (mAP_M_), and large (mAP_L_) objects, as defined by the COCO evaluation criteria.

Beyond accuracy, model efficiency is a key focus. We assess this using two metrics: the total number of trainable parameters (in millions, M) to measure the model’s spatial complexity, and the Floating Point Operations (FLOPs) (in GFLOPs) to quantify its computational complexity.

### 3.4. Implementation Details

All experiments were conducted on a Linux server equipped with eight NVIDIA RTX A6000 GPUs. The software environment was configured with Python 3.8, PyTorch 2.1.0, and MMDetection toolbox, accelerated by CUDA 11.8 and cuDNN 8.7. For our model, we employed the AdamW optimizer for 150 epochs with an initial learning rate of 0.006, a weight decay of 0.05, and a total batch size of 16 across all GPUs. For all other comparative models, the default hyperparameters from the MMDetection [28] toolbox were utilized without modification to ensure a fair comparison. The dataset was randomly partitioned into training, validation, and test sets using an 8:1:1 ratio.

### 3.5. Overview of Methods

**Figure 3:**
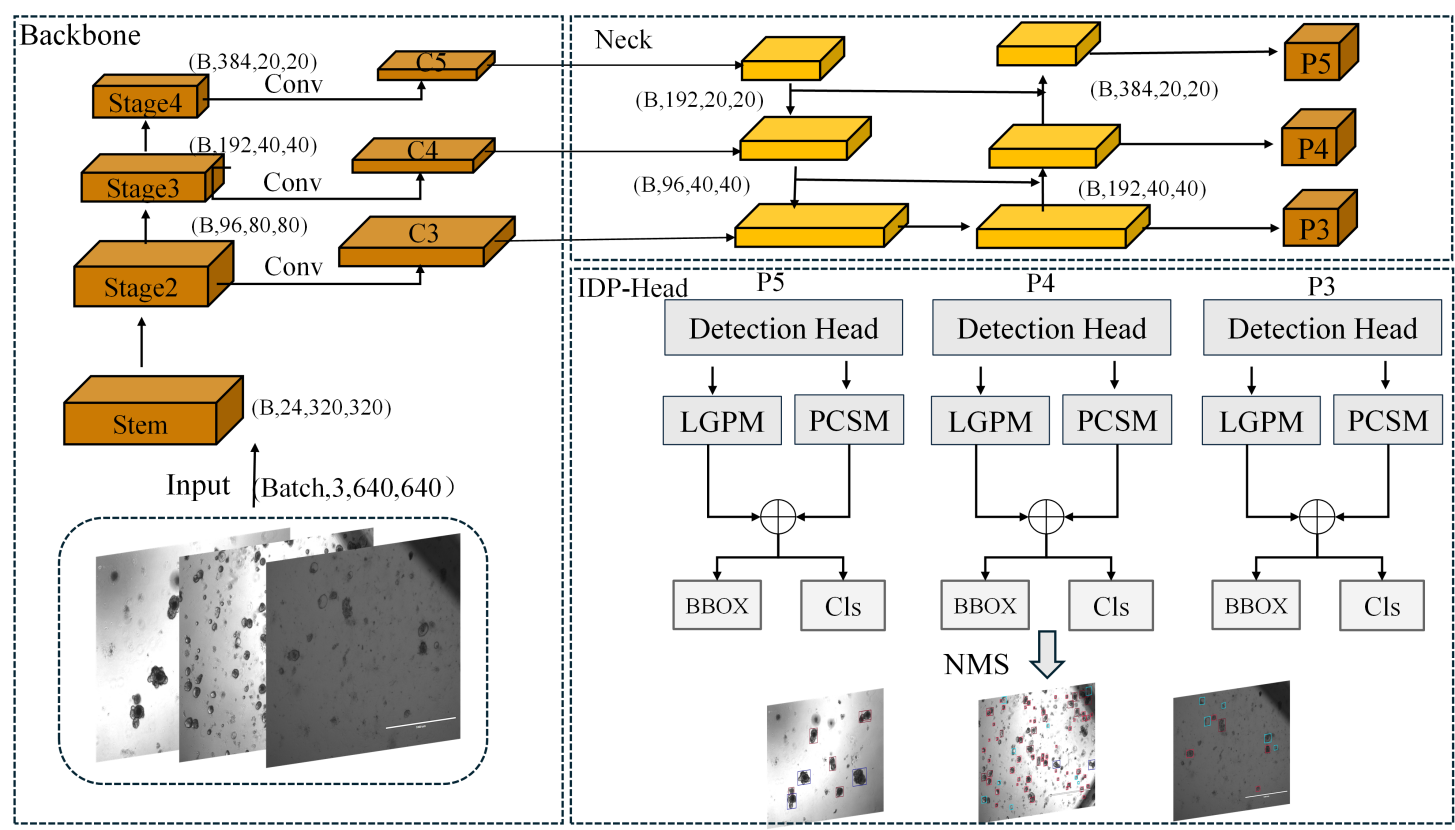
Model Architecture.

We propose Org-IDPNet, a single-stage detector for high-throughput, high-precision organoid detection, whose architecture carefully balances accuracy and real-time performance (Figure 3). The framework comprises three key components: CSPDarkNet as the backbone for feature extraction, RTMFPN as the multi-scale feature fusion hub, and our custom IDP-Head tailored to organoid characteristics. In the processing pipeline, the input bright-field image first passes through CSPDarkNet, which efficiently generates a feature pyramid embedding rich, multi-level semantic information. This pyramid is then fed into RTMFPN for cross-scale alignment and fusion, ensuring the model simultaneously captures the macro-structures of large, mature organoids and the fine details of nascent, smaller organoids. The fused feature maps are finally processed by IDP-Head, where they undergo two parallel, complementary enhancement modules: LGPM, which captures long-range dependencies and global morphology in the spatial domain, and PCSM, which models inter-channel semantic relationships in a fine-grained manner. Outputs from these two modules are merged via element-wise addition to produce enriched feature representations combining both macro- and micro-level cues, which are then passed to parallel classification and regression branches to predict organoid classes and their bounding boxes.

### 3.6. Interactive Dual-Perception Head

**Figure 4:**
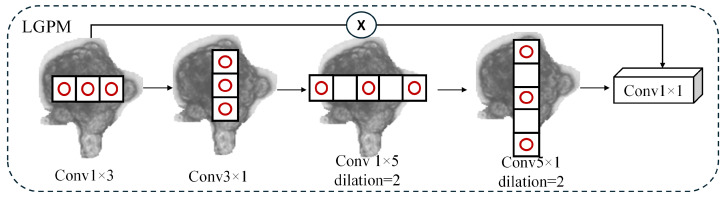
Structural Design of LGPM.

**Figure 5:**
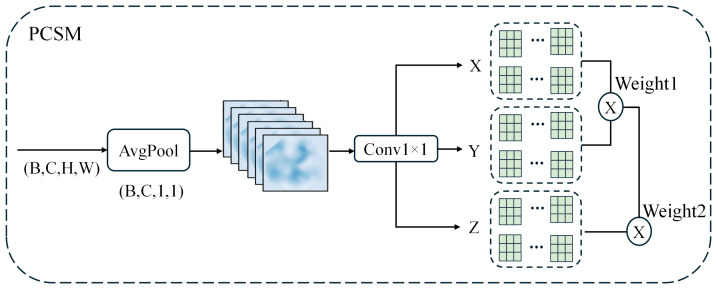
Structural Design of PCSM.

We propose IDP-Head, an architecture built upon the RTMDet framework that simultaneously captures local details and global semantic dependencies for organoid object detection. Given a multi-scale feature pyramid ${\left\{X_{i}\right\}}_{i=1}^{N}$, where each input feature map is denoted as $X\in R^{B\times C\times H\times W}$, our head consists of two parallel enhancement branches. The LGPM, which focuses on spatial structure modeling (as illustrated in Figure 4), constructs multi-scale representations from local to global by progressively enlarging the effective receptive field to capture both fine-grained textures and global contours, and by aggregating the resulting multi-scale information into spatial weights for adaptive feature modulation. In this way, the model simultaneously enhances the weak signals of small organoids while preserving the global consistency of large organoids or organoids undergoing growth and deformation. The PCSM, which models channel dependencies (as shown in Figure 5), is the parallel branch.

Their outputs are fused via element-wise addition to form a unified enhanced representation:

(6) $$F=Z_{\mathrm{LGPM}}+Z_{\mathrm{PCSM}}$$

The LGPM is designed to simulate a large receptive field using efficient depthwise convolutions. It first applies two separable convolutions along horizontal and vertical directions:

(7) $$U_{1}={\mathrm{DWConv}}_{\left(1,K\right)}\left(X\right),\;U_{2}={\mathrm{DWConv}}_{\left(K,1\right)}\left(U_{1}\right),$$

followed by dilated depthwise convolutions with the dilation rate r=2:

(8) $$U_{3}={\mathrm{DWConv}}_{(1,K),\;r=2}\left(U_{2}\right),\;U_{4}={\mathrm{DWConv}}_{(K,1),\;r=2}\left(U_{3}\right).$$

Then, 1×1 convolution and SiLU activation are used to generate a spatial attention map:

(9) $$A_{\mathrm{LGPM}}=\sigma \left({\mathrm{Conv}}_{1\times 1}\left(U_{4}\right)\right),$$

which is applied element-wise to the input:

(10) $$Z_{\mathrm{LGPM}}=X⊙A_{\mathrm{LGPM}}.$$

This path enables progressive spatial perception from local textures to global contours. Complementing this spatial enhancement, the PCSM is designed to model long-range dependencies across channels, a process it initiates with global average pooling:

(11) $$\hat{X}=\frac{1}{HW}\sum_{h=1}^{H}\sum_{w=1}^{W}X_{:,:,h,w},$$

followed by group normalization:

(12) $${\hat{X}}^{\mathrm{norm}}=\mathrm{GroupNorm}\left(\hat{X}\right).$$

The tensors *X*, *Y*, and *Z* are generated using depthwise convolutions:

(13) $$X={\mathrm{DWConv}}_{X}\left({\hat{X}}^{\mathrm{norm}}\right),\;Y={\mathrm{DWConv}}_{Y}\left({\hat{X}}^{\mathrm{norm}}\right),\;Z={\mathrm{DWConv}}_{Z}\left({\hat{X}}^{\mathrm{norm}}\right).$$

(14) $$X^{\prime },Y^{\prime },Z^{\prime }=\mathrm{Reshape}\left(X,Y,Z;\;h,d\right).$$

After reshaping into *h* attention heads, scaled dot-product attention is computed as follows:

(15) $$A1=\mathrm{Softmax}\left(\frac{X^{\prime }Y^{\prime ⊤}}{\sqrt{d}}\right),\;O=A1\cdot Z^{\prime }.$$

The output is then reshaped back, averaged spatially, and activated to generate channel attention weights:

(16) $$W_{\mathrm{PCSM}}=\sigma \left({\mathrm{Mean}}_{h,w}\left({\mathrm{Rearrange}}^{-1}\left(O\right)\right)\right),$$

(17) $$Z_{\mathrm{PCSM}}=X⊙W_{\mathrm{PCSM}}.$$

Multi-branch Prediction

The fused feature *F* is passed into parallel classification and regression branches. The classification output is defined as

(18) $$P_{\mathrm{cls}}\in R^{B\times (A\cdot C)\times H\times W},$$

and the bounding box regression branch produces

(19) $$P_{\mathrm{reg}}\in R^{B\times (A\cdot 4)\times H\times W}.$$

Here, *B* is the batch size, *A* is the number of anchors per location, *C* is the number of classes, and *H* and *W* are the spatial dimensions (height and width) of the feature map.

This interactive dual-pathway design enables IDP-Head to effectively combine structural context and channel-wise semantics, improving detection accuracy and robustness for morphologically complex organoid targets.

## 4. Experimental Results

This subsection details the comparative experiments and ablation studies conducted on the TelluOrg and MouseOrg datasets, and presents the experimental results based on key evaluation metrics.

### 4.1. Comparative Experiments on MouseOrg

To validate the performance of the proposed Org-IDPNet in a basic binary detection task, we compared it against several state-of-the-art object detectors. The quantitative results are summarized in Table 1. Org-IDPNet achieves the best performance across all key precision metrics, with an mAP of 76.7%, substantially outperforming all competing methods, including CO-DINO (74.5%) and YOLOv11 (74.3%). Under the more stringent localization criterion mAP_75_, our method also ranks first, demonstrating its superior bounding box regression capability. Crucially, compared to our baseline RTMDet-L, Org-IDPNet attains a net gain of +9.7 percentage points in mAP, while simultaneously reducing the model size and computational cost. This result convincingly confirms that the proposed IDP-Head can efficiently enhance the detector’s representational power for complex organoid features. Moreover, Org-IDPNet achieves the highest detection accuracy at small, medium, and large scales (mAP*_s_*/mAP*_m_*/mAP*_l_*), highlighting its strong multi-scale adaptability.

**Figure 6:**
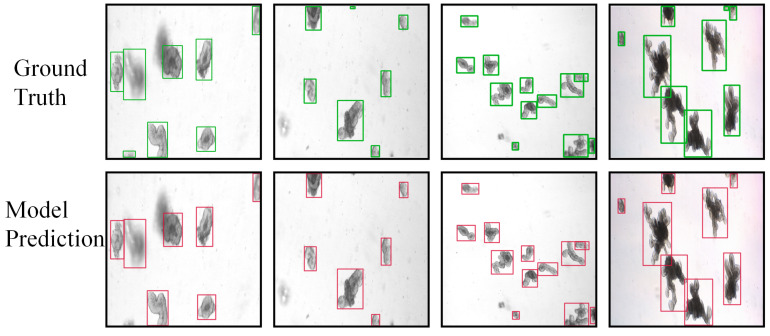
Comparison Between Ground Truth and Model Predictions.

In addition to numerical comparisons, we further illustrate the practical detection performance of Org-IDPNet through visualizations in Figure 6. The figure juxtaposes representative bright-field images of organoids, corresponding ground-truth annotations, and our model’s predicted bounding boxes. As shown, Org-IDPNet accurately localizes the vast majority of organoids in the field of view, with high overlap between predictions and ground truth. Even for organoids exhibiting irregular shapes, blurred boundaries, or close proximity, our model reliably delineates precise contours, yielding minimal false negatives and false positives. These qualitative results provide direct evidence of the model’s robust capability for high-precision organoid detection in complex bright-field imagery.

### 4.2. Comparative Experiments on TelluOrg

In the second set of comparative experiments conducted on the TelluOrg dataset, we faced significant challenges due to its highly imbalanced class distribution and frequent overlaps among targets. These characteristics impose more stringent requirements on the robustness and generalization capability of detection models. As shown in Table 2, we performed a comprehensive evaluation of various state-of-the-art detectors on this dataset. The experimental results clearly demonstrate that our method consistently achieved the best performance across all critical accuracy metrics. Specifically, it attained a mean Average Precision (mAP) of 66.7%, markedly surpassing all compared approaches, including the strong baseline RTMDet-L (63.7%) and the advanced CO-DINO (60.6%). These findings further validate the outstanding balance of our method between accuracy and efficiency, as it achieves the highest precision while maintaining relatively low model complexity and computational overhead.

This study conducts a detailed comparison of Org-IDPNet against EfficientDet, DINO-4scale, and the baseline RTMDet-L model, evaluating per-category mean Average Precision (mAP) on organoid images (in Table 3). The proposed Org-IDPNet, as an advanced single-stage detector, consistently outperforms its counterparts: it surpasses the strong single-stage baseline RTMDet-L by 4.9 % and 4.6 % mAP on the “Spheroid” and “Organoid0” categories, respectively, and significantly exceeds the classic EfficientDet. Moreover, when compared with the Transformer-based DINO-4scale—whose architecture has exhibited excellent performance in general object detection—Org-IDPNet demonstrates even greater advantages on specialized organoid imagery, with DINO’s metrics falling substantially behind. These comprehensive, category-wise results convincingly demonstrate that Org-IDPNet is better suited than other leading single-stage and Transformer-based detectors for the complex task of organoid detection.

**Figure 7:**
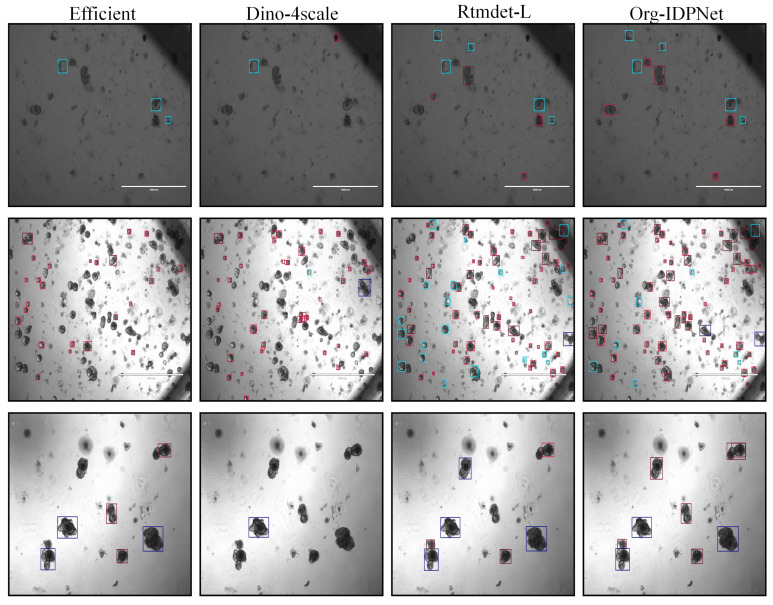
Comparison of Detection Results Across Different Models.

The quantitative advantages of our model are further corroborated by qualitative visualization results. As illustrated in Figure 7, we compared the prediction maps of Org-IDPNet against those of EfficientDet, DINO-4scale, and the baseline RTMDet-L on the same complex scene. It is clearly observable that EfficientDet suffers from numerous missed detections (false negatives), while DINO-4scale struggles with irregularly shaped organoids. The baseline model, RTMDet-L, exhibits imprecise localization for certain organoids with ambiguous boundaries. In contrast, our Org-IDPNet demonstrates superior detection performance. Not only does it successfully identify the majority of organoids in the field of view, including difficult samples missed by other models, but its generated bounding boxes also achieve the tightest fit to the actual object contours.

### 4.3. Ablation Study

Ablation studies were conducted on two datasets to dissect the individual contributions and synergistic effects of the proposed LGPM and PCSM modules. As shown in Table 4, integrating either module alone enhances the baseline performance, validating their independent effectiveness.

Further analysis reveals that the LGPM module significantly enhances the precision of bounding box regression through its localization-guided multi-scale modeling, an improvement especially prominent for the high-threshold metric mAP75, which is more sensitive to localization accuracy, yielding gains of +1.8% on the MouseOrg dataset and +1.4% on the TelluOrg dataset. In contrast, the PCSM module, by employing a channel-spatial progressive enhancement strategy, effectively boosts feature discriminability and recall; consequently, its advantages are more pronounced in terms of improvement in the overall mAP and the low-threshold metric mAP50, with recorded gains of +2.2% and +1.1% on MouseOrg, respectively. When used in combination, they exhibit a significant complementary effect, achieving performance growth across all evaluation metrics that surpasses the linear superposition of their individual contributions, thus demonstrating a synergistic “1 + 1 > 2” advantage. Mechanistically, this synergistic performance enhancement stems from LGPM’s effective suppression of localization error and PCSM’s significant mitigation of classification/miss error, providing a solid and interpretable theoretical foundation for the exceptional performance of the combined model.

## 5. Discussion

Beyond the aggregate mAP metric, we conducted a comprehensive and fine-grained analysis to better understand the model’s performance in the organoid detection task. This investigation moves beyond quantifying correct predictions to probe the specific nature and sources of error. To this end, three complementary indicators were jointly assessed: the confusion matrix, precision, and recall. The confusion matrix offers a qualitative perspective, visually revealing inter-class misclassifications and pinpointing weaknesses in distinguishing morphologically similar organoids. Concurrently, precision and recall provide quantitative insights by measuring the accuracy of positive predictions and the completeness of detections, respectively. By integrating these metrics, we achieve a holistic evaluation of the model’s behavior, elucidate the trade-off between its predictive accuracy and detection coverage, and arrive at a more robust judgment of its reliability for practical applications.

### 5.1. Confusion Matrix Analysis

**Figure 8:**
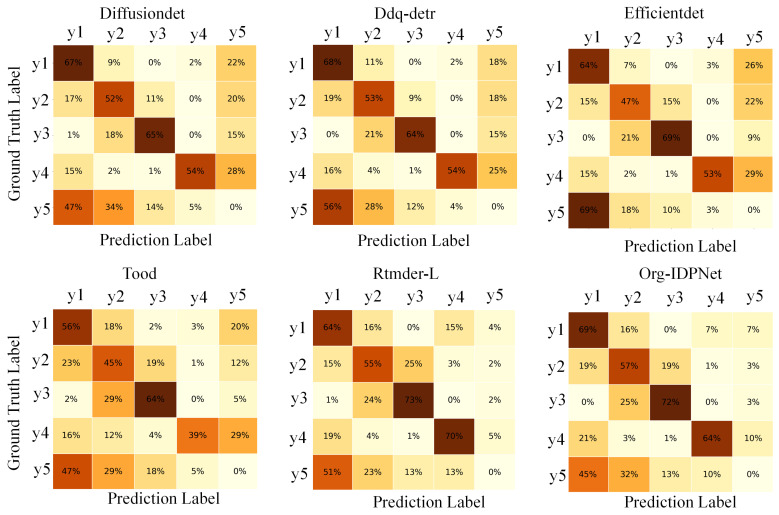
Confusion Matrices of Different Models, With Darker Colors Indicating Better Performance.

As illustrated in Figure 8, we analyzed the normalized confusion matrix, where labels y1 through y5 represent organoid0, organoid1, organoid3, spheroid, and background, respectively. The results clearly demonstrate that the proposed Org-IDPNet exhibits a significant advantage in class-wise discrimination. It achieves the highest classification accuracy across several key categories—most notably y1 (69%) and y3 (72%)—among all evaluated models, while also exhibiting a superior ability to suppress inter-class confusion.

A prominent example of this strength is the model’s performance on background classification: unlike other models that frequently misclassify a large portion of background (y5) as foreground objects, Org-IDPNet restricts this false positive rate to just 32%, the lowest among all competitors. Furthermore, in the case of visually and structurally similar classes such as y1 and y2, Org-IDPNet demonstrates enhanced robustness and discrimination capacity.

These qualitative findings are well aligned with quantitative metrics, jointly confirming the outstanding performance of the Org-IDPNet architecture in improving organoid detection accuracy and reducing misclassification between classes.

### 5.2. Precision Analysis

The class-wise precision comparison, as shown in Table 5, further validates the superiority of Org-IDPNet. Our model consistently outperforms both the baseline RTMDet-L and the state-of-the-art detector TOOD across all categories, achieving notably higher precision scores. Most strikingly, Org-IDPNet significantly improves precision for challenging categories such as “spheroid”, increasing it from 0.351 (RTMDet-L) to 0.549—an impressive gain of nearly 20 percentage points. Similarly, for ”organoid0”, precision improves from 0.484 to 0.598. Compared to TOOD, Org-IDPNet also demonstrates a clear advantage, with a 7-point gain in “organoid3” (0.551 vs 0.483). These consistent improvements highlight Org-IDPNet’s stronger discriminative capability, especially in difficult cases, and underscore its ability to make more confident and accurate positive predictions while effectively reducing false positives.

### 5.3. Recall Analysis

**Figure 9:**
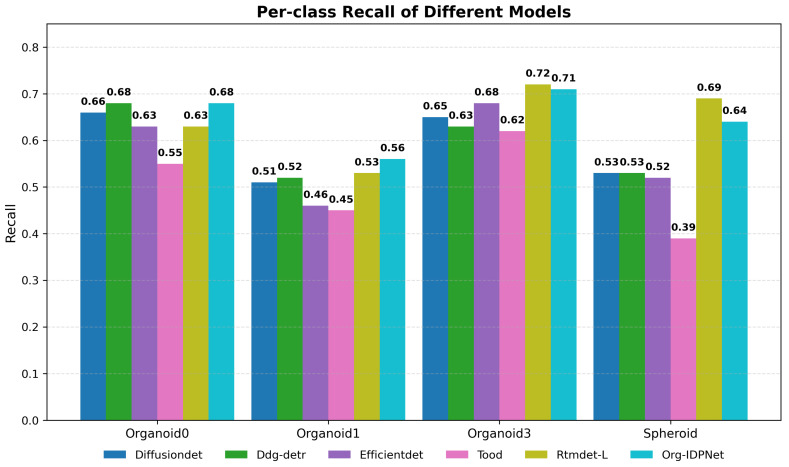
Per-class Recall.

In terms of recall performance, Org-IDPNet also demonstrates highly competitive results. As shown in Figure 9, our model achieves the highest recall among all compared methods for “organoid0” (0.68) and “organoid1” (0.56), effectively reducing the missed detection rate. Although the baseline model RTMDet-L achieves slightly higher recall in “organoid3” and “spheroid”, its performance on other categories—such as only 0.53 for organoid1—is suboptimal, indicating weaker overall consistency. In contrast, Org-IDPNet maintains high recall across all categories, highlighting its superior and balanced capability to retrieve true positives. Moreover, compared with Transformer-based architectures such as Ddq-DETR and DiffusionDet, as well as classical detectors like EfficientDet and TOOD, Org-IDPNet consistently achieves better recall. These results strongly suggest that the proposed model not only excels in feature extraction, but also enables more comprehensive and reliable detection of organoid targets within the image.

## 6. Conclusions

This study addresses the challenge of accurate detection in automated organoid analysis, which is often hindered by the morphological diversity, dense overlaps, and ambiguous boundaries of organoid structures. To this end, we propose Org-IDPNet, a novel and efficient single-stage detection framework specifically tailored for this task. At the core of Org-IDPNet is IDP-Head, which integrates two complementary modules: the LGPM captures rich multi-scale spatial structures and contextual information through parallel pathways, while the PCSM models long-range dependencies across feature channels. Together, these modules enhance the model’s representational and discriminative power.

Comprehensive experiments validate the effectiveness of our approach. First, ablation studies not only confirm the individual contributions of the LGPM and PCSM, but also highlight their strong synergistic effect, where their combination yields performance gains greater than the sum of their parts. Second, in extensive comparisons with leading detectors—including the strong baseline Rtmdet-L, the classical EfficientDet, and the Transformer-based Dino-4scale—Org-IDPNet achieves superior performance across all major evaluation metrics, including mAP, mAP50, and mAP75.

Further class-wise analyses reveal the model’s inherent advantages. Precision and recall metrics jointly demonstrate that Org-IDPNet effectively balances false positive suppression with minimized missed detections. Qualitative results, including visualizations and confusion matrix analysis, provide intuitive evidence that the model can reliably distinguish morphologically similar classes and accurately detect challenging samples that are often misclassified by other methods. Although the LGPM and PCSM strengthen multi-scale and channel-dependency modeling, the network can still miss or mis-detect targets in scenarios with very small objects, severe occlusion, or extreme crowding. When multiple organoids are highly adherent/closely connected, the model may also collapse them into a single instance. In addition, the current study focuses on detection only, and does not yet provide an end-to-end solution for fine-grained instance segmentation, morphological quantification, or longitudinal spatiotemporal analysis of organoid growth.

Looking ahead, we plan to extend this work in several directions. First, we aim to expand the current 2D detection framework [42] into a 3D paradigm, enabling more comprehensive segmentation of organoid structures and tracking of their dynamic growth processes. Second, we will explore transferring the core design of Org-IDPNet to broader biomedical imaging applications, such as nuclear detection in histopathological slides [43] or tumor region identification. Finally, efforts will be made toward model lightweighting and deployment optimization, facilitating seamless integration into existing microscopic imaging and analysis platforms, and ultimately advancing the automation of life science research.

## Figures and Tables

**Table 1:** Comparative Experimental Results on MouseOrg.

Methods	Evaluation Metrics							
	mAP	mAP50	mAP75	mAP-s	mAP-m	mAP-l	Parameters	Flops
Yolof [29]	0.285	0.634	0.209	0.128	0.405	0.403	42.339 M	61.222 G
Vfnet [30]	0.366	0.669	0.368	0.359	0.376	0.410	32.709 M	118 G
Tood [31]	0.538	0.861	0.607	0.494	0.585	0.525	32.018 M	123 G
Sparse-RCNN [32]	0.134	0.302	0.106	0.117	0.174	0.089	106 M	97.63 G
Faster-RCNN	0.179	0.432	0.104	0.103	0.267	0.129	41.348 M	134 G
EfficientDet [33]	0.607	0.862	0.671	0.454	0.717	0.688	3.828 M	2.303 G
DINO-4scale [34]	0.487	0.723	0.609	0.455	0.586	0.590	47.54 M	179 G
DiffusionDet [35]	0.704	0.887	0.784	0.601	0.800	0.743	–	–
Deformable-DETR [36]	0.425	0.762	0.437	0.317	0.519	0.470	40.099 M	123 G
DDQ-DETR-4scale [37]	0.412	0.724	0.457	0.367	0.498	0.426	–	–
Ddod [38]	0.473	0.846	0.504	0.462	0.498	0.421	32.196 M	111 G
Co-DETR-5scale [39]	0.745	0.917	0.857	0.641	0.853	0.810	64.455 M	–
CenterNet [40]	0.391	0.745	0.367	0.420	0.384	0.324	32.111 M	123 G
YOLO V11 [41]	0.743	0.934	0.851	0.618	0.838	0.796	2.58 M	6.3 G
RTMDet-L	0.670	0.894	0.785	0.557	0.767	0.765	52.255 M	79.951 G
Org-IDPNet (Ours)	0.767	0.941	0.874	0.638	0.855	0.849	5.548 M	8.112 G

**Table 2:** Comparative Experimental Results on TelluOrg.

Methods	Evaluation Metrics							
	mAP	mAP50	mAP75	mAP-s	mAP-m	mAP-l	Parameters	FLOPs
YOLOF	0.124	0.267	0.099	0.009	0.087	0.151	42.409 M	83.32 G
VFNet	0.105	0.214	0.091	0.028	0.087	0.083	32.716 M	161 G
TOOD	0.394	0.605	0.441	0.153	0.405	0.346	32.025 M	168 G
Sparse-RCNN	0.106	0.220	0.091	0.034	0.090	0.090	106 M	130 G
Faster-RCNN	0.109	0.256	0.074	0.039	0.095	0.137	41.364 M	178 G
EfficientDet	0.479	0.703	0.534	0.131	0.471	0.674	3.83 M	2.313 G
DINO-4scale	0.367	0.530	0.425	0.183	0.374	0.435	47.546 M	235 G
DiffusionDet	0.489	0.699	0.553	0.184	0.501	0.494	–	–
Deformable-DETR	0.322	0.566	0.325	0.110	0.312	0.449	40.099 M	165 G
DDQ-DETR-4scale	0.519	0.718	0.599	0.196	0.530	0.590	–	–
DDOD	0.255	0.432	0.280	0.099	0.232	0.229	32.203 M	151 G
Co-DINO-5scale	0.606	0.788	0.704	0.258	0.615	0.666	64.483 M	–
CenterNet	0.202	0.376	0.197	0.077	0.206	0.170	32.118 M	167 G
YOLO V11	0.595	0.785	0.689	0.224	0.610	0.592	2.583 M	6.3 G
RTMDet-L	0.637	0.833	0.737	0.253	0.645	0.659	52.257 M	79.958 G
Org-IDPNet (Ours)	0.667	0.860	0.775	0.255	0.684	0.736	5.549 M	8.115 G

**Table 3:** Comparison Between EfficientDet, DINO-4scale, RTMDet-L, and Org-IDPNet on Different Categories.

	EfficientDet			DINO-4scale		
Category	mAP	mAP50	mAP75	mAP	mAP50	mAP75
Organoid0	0.360	0.632	0.358	0.394	0.589	0.476
Organoid1	0.405	0.612	0.451	0.307	0.446	0.355
Organoid3	0.601	0.847	0.705	0.405	0.598	0.428
Spheroid	0.549	0.722	0.624	0.370	0.486	0.438
	RTmdet-L			Org-IDPNet (Ours)		
Category	mAP	mAP50	mAP75	mAP	mAP50	mAP75
Organoid0	0.566	0.811	0.655	0.612	0.847	0.716
Organoid1	0.627	0.827	0.725	0.644	0.847	0.744
Organoid3	0.702	0.880	0.806	0.714	0.895	0.833
Spheroid	0.651	0.815	0.762	0.700	0.852	0.806

**Table 4:** Ablation Studies on Different Datasets.

	MouseOrg			TelluOrg		
Method	mAP	mAP50	mAP75	mAP	mAP50	mAP75
base	0.734	0.92	0.847	0.642	0.836	0.744
base + LGPM	0.753	0.933	0.865	0.655	0.85	0.758
base + PCSM	0.756	0.931	0.862	0.65	0.846	0.762
base + LGPM + PCSM	0.767	0.941	0.874	0.667	0.86	0.775

**Table 5:** Per-class Precision.

Method	organoid0	organoid1	organoid3	Spheroid
Tood	0.505	0.400	0.483	0.593
RTMDet-L	0.484	0.458	0.456	0.351
Org-IDPNet	0.598	0.507	0.551	0.549

## Data Availability

Data requests can be emailed to the corresponding author’s email address.
